# Hypoxia-regulated target genes implicated in tumor metastasis

**DOI:** 10.1186/1423-0127-19-102

**Published:** 2012-12-14

**Authors:** Ya-Ping Tsai, Kou-Juey Wu

**Affiliations:** 1 Institute of Biochemistry & Molecular Biology, National Yang-Ming University, No.155, Li-Nong St., Sec.2, Peitou, Taipei 112, Taiwan; 2 Head and Neck Cancer Research Program, Cancer Research Center, National Yang-Ming University, No.155, Li-Nong St., Sec.2, Peitou, Taipei 112, Taiwan; 3 Genome Research Center, National Yang-Ming University, Taipei 112, Taiwan

**Keywords:** Hypoxia, Hypoxia-inducible factor, Target genes, Metastasis, Epithelial-mesenchymal transition

## Abstract

Hypoxia is an important microenvironmental factor that induces cancer metastasis. Hypoxia/hypoxia-inducible factor-1α (HIF-1α) regulates many important steps of the metastatic processes, especially epithelial-mesenchymal transition (EMT) that is one of the crucial mechanisms to cause early stage of tumor metastasis. To have a better understanding of the mechanism of hypoxia-regulated metastasis, various hypoxia/HIF-1α-regulated target genes are categorized into different classes including transcription factors, histone modifiers, enzymes, receptors, kinases, small GTPases, transporters, adhesion molecules, surface molecules, membrane proteins, and microRNAs. Different roles of these target genes are described with regards to their relationship to hypoxia-induced metastasis. We hope that this review will provide a framework for further exploration of hypoxia/HIF-1α-regulated target genes and a comprehensive view of the metastatic picture induced by hypoxia.

## Review

### Introduction

Hypoxia is a critical microenvironmental factor that is demonstrated to induce tumor metastasis [1-5]. Hypoxia has profound effects in metabolism, angiogenesis, innate immunity, and stemness induction [1]. The effects of hypoxia are usually mediated by hypoxia-inducible factors (HIFs), i.e. HIF-1α and HIF-2α [1−4]. HIFs heterodimerize with a common partner, HIF-1β (or aryl hydrocarbon receptor nuclear translocator (ARNT)), to regulate downstream target gene expression through a hypoxia-response element (HRE) [1]. The consensus of HRE is 5’-RCGTG-3’, although other sequences were shown to respond to HIFs [3,4]. An ancillary sequence was shown to couple with the canonical HRE to better respond to the binding of HIFs [6]. Although microarray and genomics approaches were performed to identify various downstream targets of HIFs, these targets only represent a small subset of targets reported in the literature [7-10]. More than one thousand target genes were reported to be regulated by HIFs to mediate the effects induced by hypoxia [3,4]. Although most target genes were identified through their regulation by HIF-1α, other targets could be solely regulated by HIF-2α [11]. However, most targets are usually regulated by both HIF-1α and HIF-2α [11]. Opposing activities between HIF-1α and HIF-2α could occur [11]. Cancer metastasis is the most significant etiology to cause patient death of cancer patients [12]. Different steps are proposed for a tumor cell to become metastatic including epithelial-mesenchymal transition (EMT), extracellular matrix modulation, intravasation, circulation, extravasation, homing and the premetastatic niche, and organotropic colonization [5,12]. Due to the causal role of hypoxia in the induction of metastasis [1-5], it will be important to assign the role of these hypoxia-regulated targets according to the various steps of metastasis. In this review, we will focus on the hypoxia/HIF-1α-regulated target genes that are shown to modulate tumor migration, invasion, and metastatic property. These target genes are divided into various categories including transcription factors, chromatin modifiers, enzymes, receptors, small GTPases, transporters, adhesion molecules, surface molecules, membrane proteins, and microRNAs. We hope that this review will provide a framework of hypoxia-regulated targets and further elucidate the molecular mechanisms to induce metastasis under hypoxic condition.

### Transcription factor and histone modifiers

One well-studied aspect of hypoxia-induced metastasis is that hypoxia induces various transcriptional regulators (also called EMT regulators) to cause EMT [13-16]. The EMT regulators including Twist1, Snail, Slug, ZEB1, ZEB2, and E12/E47 have been shown to be either directly or indirectly regulated by HIF-1α [17]. These EMT regulators subsequently bind to the promoters of EMT marker genes including E-cadherin, vimentin, and N-cadherin to mediate EMT [16,17]. The EMT regulator Snail is a good example of indirect regulation by hypoxia through the lysyl oxidase-like 2 enzyme [18]. The nuclear localization of Snail could also be regulated by ERβ (estrogen receptor β) to impede EMT in prostate cancer cells [19]. Other transcription factors are also regulated by HIF-1α to contribute to metastasis. For example, inhibitor of differentiation/DNA binding 2 (ID2) is regulated by HIF-1α to cause dedifferentiation of neuroblastoma cells, which leads to less mature and more aggressive tumors [20]. Hypoxia upregulates the expression of Ewing Sarcoma-Friend leukemia virus integration 1 (EWS-FLI1) chimeric protein to modulate its transcriptional signature and increase the malignant properties of Ewing’s sarcoma cells [21]. Hypoxia activates β-catenin in hepatocellular carcinoma cells to induce EMT and enhance their metastatic potential [22]. Inverse correlation between the expression of caudal type homeobox 2 (CDX2), a homeodomain protein, and HIF-1α is observed in colorectal cancer samples [23]. Finally, hypoxia converts the inhibitor function of SMAD family member 7 (SMAD7, an inhibitor of the TGF-β signaling pathway) into a promoter of cell invasion [24]. In addition to transcriptional factors, chromatin modifiers could also be regulated by hypoxia. Induction of histone lysine-specific demethylase 4B (KDM4B, JMJD2B) correlates with invasion and advanced clinical stage in colorectal cancers [25]. JMJD2B also plays a central role in gastric cancer cell growth [26]. JMJD2C (KDM4C) is induced by HIF-1α to mediate epigenetic regulation of HIF-1α downstream genes involved in metabolic reprogramming and lung metastasis of breast cancer [27]. The histone methyltransferase mixed lineage leukemia 1 (MLL1) is induced by hypoxia to enhance hypoxic response [28]. All these results demonstrated that hypoxia/HIF-1α regulates both transcription factors and chromatin modifiers to induce metastasis in an EMT-dependent or independent manner.

### Enzymes that are regulated by hypoxia/HIF-1α

Hypoxia/HIF-1α is originally shown to regulate the expression of metalloproteases including matrix metalloprotease-1 (MMP1) and MMP3 to induce metastasis [29]. In contrast, HIF-2α activates the expression of membrane type-1 matrix metalloproteinase (MT1-MMP) in von Hippel-Lindau renal cell carcinoma [29,30]. The most significant enzyme that is regulated by hypoxia to cause metastasis is lysyl oxidase (LOX) [31]. Secreted LOX is required for adhesion interactions necessary for migration through focal adhesion kinase activity and cell to matrix adhesion [31]. Lysyl oxidase is also a crucial mediator of bone marrow cell recruitment to form the premetastatic niche through crosslinking collagen IV and recruiting CD11b myeloid cells [32]. Other lysyl oxidase-like enzymes that are regulated by HIF-1α also play an important role in the formation of breast cancer metastatic niche [33]. Loss of programmed cell death 4 (Pdcd4), a tumor suppressor, increases the expression of lysyl oxidase and hypoxia-induced breast cancer cell invasion through a HIF-1-independent mechanism [34]. A disintegrin and metalloproteinase with thrombospondin motifs 1 (ADAMTS1) is a hypoxic early response gene in endothelial cells that promotes migration under hypoxia [35]. Hypoxia activates the expression of angiotensin converting enzyme (ACE) and inhibits ACE2 expression in human pulmonary smooth muscle cells [36]. Sulfatase 1 (Hsulf-1) modulates the sulfation state of heparin sulfate proteoglycans and hypoxia represses Hsulf-1 expression that leads to increased bFGF signaling and the promotion of migration and invasion of breast cancer cells [37]. Hypoxia promotes isocitrate dehydrogenase (IDH2)-dependent carboxylation of α-ketoglutarate to support cell growth and viability [38]. HIF-1α regulates xeroderma pigmentosum, complementation group A (XPA) expression and contributed to cisplatin resistance in lung cancer [39]. The results described above indicate that various enzymes are regulated by hypoxia/HIF-1α to regulate the metastatic activity of tumor cells.

### Receptors, kinases, small GTPases, and transporters

Various receptors, receptor-activated kinases, small GTPases, and transporters are regulated by hypoxia/HIF-1α to play a significant role in cancer metastasis. For example, chemokine receptor 4 (CXCR4) is one of the early identified hypoxia/HIF-1α target genes that mediates hypoxia-induced angiogenesis and metastasis [40]. Similar regulation of another chemokine receptor, chemokine (C-X3-C motif) receptor 1 (CX3CR1), is also shown in prostate cancer and pancreatic ductal adenocarcinoma cells to mediate their migration and invasion [41,42]. Notch signaling has recently been shown to be required for hypoxia-induced EMT, tumor cell migration and invasion through activation of Snail [43]. The urokinase-type plasminogen activator receptor (uPAR) is activated by HIF-1α and is crucial for hypoxia-induced metastasis [44]. Similarly, plasminogen activator inhibitor-1 (PAI-1), a factor related to the uPAR system that predicts poor prognosis in many cancers, is regulated by hypoxia through the cooperative functions of HIF-1α, Egr-1, and C/EBPα [45]. The 67-kDa laminin receptor was shown to be activated by hypoxia to promote gastric cancer metastasis through increasing uPA and MMP9 expession [46]. Toll-like receptor 4 (TLR4) is induced by HIF-1α to promote pancreatic tumor cell growth [47]. C-Met protooncogene expression is regulated by hypoxia to enhance scatter factor/hepatocyte growth factor (SF/HGF)-induced cell migration and invasion in glioma and trophoblast cells [48,49]. Pericyte depletion triggers hypoxia-induced EMT and metastasis through the c-Met signaling [50]. RON tyrosine kinase is a direct target of HIF-1α and mediates invasion of breast carcinoma cells [51]. Nitric oxide-induced macrophage migration is mediated through HIF-1α-regulated small GTPases Cdc42 and Rac1 [52]. RhoE activation by HIF-1α mediates hypoxia-induced EMT of gastric cancer cells [53]. Insulin receptor substrate 2 (IRS-2) is regulated by hypoxia to promote breast carcinoma cell survival and invasion [54]. Different transporters including glucose transporter type 1 (glut-1) and multidrug resistance protein 1 (MDR1) are regulated by hypoxia/HIF-1α and implicated in the metastatic processes [55,56]. A truncated form of the voltage-dependent anion channel 1 (VDAC1) is induced by HIF-1α to promote cancer cell survival and correlates with chemotherapy resistance in lung cancer patients [57].

### Adhesion molecules, surface molecules, membrane proteins, and various proteins implicated in hypoxia-induced metastasis

Various adhesion or surface molecules, membrane proteins, and miscellaneous proteins are regulated by hypoxia/HIF-1α and involved in hypoxia-induced metastasis. For breast cancer, angiopoietin-like 4 (ANGPTL4) and L1 cell adhesion molecule (L1CAM) are the two important molecules that mediate vascular metastasis of hypoxic breast cancer cells to the lungs [58]. ANGPTL4 inhibits EC (endothelial cell)-EC interactions, whereas L1CAM promotes the adherence of breast cancer cells to ECs [58]. HIF-1α mediates anoikis resistance through suppression of α5 integrin [59]. CD151, a member of the tetraspanin family that plays a role in cell adhesion and motility, is repressed by hypoxia to regulate the detachment of colorectal cancer cells from the primary site and homing in the second site [60]. CD24, a cancer stem cell-associated membrane protein, is an effector of HIF-1α-driven primary tumor growth and metastasis [61]. CD147 is induced by HIF-1α and Sp1 to promote glycolysis and tumor progression in epithelial solid tumors [62]. Galectin-1, one of the important lectins contributing to malignant tumor formation, is induced by HIF-1α to mediate migration and invasion of colorectal cancer cells [63]. MUC1, an O-glycoprotein membrane-bound mucin, is a HIF-1α target that plays a role in regulating the migration and invasive property of renal cell carcinoma cells [64]. Semaphorin 4D is induced by HIF-1α to promote angiogenesis and enhance tumor invasive growth of head and neck cancers [65]. Caveolin-1, an essential structural constituent of caveolae on the cell membrane involved in endocytosis, is upregulated by HIF-1α to promote ligand-independent EGF receptor signaling and increase the cell proliferative, migratory, and invasive capacities [66]. Human enhancer of filamentation 1 (HEF1, NEDD9, Cas-L), a scaffold protein implicated in cellular attachment and motility, is a mediator of HIF-1α-induced migration in colorectal carcinoma cells [67]. Liprin-α4 is a HIF-1α-regulated cytoplasmic protein required for maintenance of cell-cell contacts in renal cell carcinoma [68]. Two matricellular proteins CYR61 (CCN1) and NOV (CCN3) are regulated by HIF-1α and TGF-β3 in human trophoblasts and increase their migration and invasion [69]. Prostate specific antigen (PSA), one of the androgen receptor target genes involved in tumor invasion, is induced by HIF-1α in prostate cancers [70]. Finally, proteomics experiments identify two HIF-1-inducible targets (S100A4, CapG) and S100A4 is involved in migration and invasion of cancer cells [71]. It is obvious that various types of proteins can play a significant role in mediating hypoxia-induced migration and invasive property of cancer cells.

### microRNA targets

Various microRNAs are shown to be regulated by hypoxia/HIF-1α. A recent review describing the various microRNAs regulated by hypoxia provides a concise summary of the roles of these microRNAs in metabolism, DNA damage response, and angiogenesis [72]. However, the role of these microRNAs in metastasis was not elucidated in this review. Among the microRNAs regulated by hypoxia, miR-210 is the critical microRNA implicated in tumor initiation and metastatic potential by targeting different downstream molecules including genes expressed under normoxia and vacuole membrane protein 1 (VMP1) [73,74]. MiR-210 is proposed to be a micromanager of the hypoxia pathway [75]. Reversion-inducing-cysteine-rich protein with kazal motifs (RECK) is regulated by both hypoxia and Ras-signaling pathways through three groups of microRNAs (miR-15b/16, miR-21, and miR-372/373) [76]. Hypoxia represses miR-34a that targets the Notch signaling pathway and promotes EMT in tubular epithelial cells [77]. HIF-α downregulates miR-17/20a that targets p21 and signal transducer and activator of transcription 3 (STAT3) and inhibited AML cell differentiation [78]. Finally, hypoxia-induced miR-103/107 targets the tumor suppressors death-associated protein kinase (DAPK) and Kruppel-like factor 4 (KLF4) to promote metastasis of colorectal cancer [79]. MiR-107 is also induced by p53 to repress the expression of HIF-1β and suppress tumor growth and angiogenesis [80]. Recent deep sequencing experiments to search for microRNAs regulated by hypoxia resulted in the discovery of known and novel microRNAs [81]. MiR-210 is still the prominent microRNA that was identified under hypoxia together with other known microRNAs (e.g. miR-126) and novel microRNAs. The role of these newly discovered microRNAs in metastasis remains to be determined.

## Conclusions

Hypoxia is one of the most important environmental factors that induce cancer metastasis. Hypoxia-regulated biological effects have been extensively investigated. It is obvious that hypoxia regulates key steps of the metastatic process. Through outlining various targets regulated by hypoxia/HIF-1α to mediate tumor metastasis, the review wishes to provide a comprehensive guide according to the various categories described. A summary of all these targets is listed in Table 1. Although angiogenesis is regulated by hypoxia and can contribute to tumor metastasis [1,2], the review only focuses on the targets related to the various steps controlling metastasis [5]. In addition, various targets regulated by hypoxia/HIF-1α to mediate other biological effects such as metabolism may also contribute to metastasis [1,2]. The functions mediated by these targets related to metabolism need to be verified using the *in vitro* and *in vivo* metastatic assays to demonstrate their contribution to tumor metastatic activity. The linkage between metabolism and tumor metastasis remains to be further explored. The concept will also apply to other hypoxia/HIF-1α-regulated targets that appear not to be directly regulating metastasis.

**Table 1:** Classification of the hypoxia-regulated genes

**Biological function**	**Gene symbol/ alias**	**References**
Transcription factors	Twist1, Snail, Slug, ZEB1, ZEB2, E12/E47, ID2,EWS-FLI1, β-catenin, CDX2, SMAD7	[13-16,20-24]
Histone modifiers	JMJD2B, JMJD2C, MLL1	[25-28]
Enzymes	MMP1, MMP3, MT1-MMP, LOX, ADAMTS1, ACE, ACE2, Hsulf-1, IDH2, XPA	[29-33,35-39]
Receptors, receptor-associated kinase	CXCR4, CX3CR1, Notch, uPAR, PAI-1, 67-kDa laminin receptor, TLR4, c-Met, RON tyrosine kinase	[40-51]
small GTPases, intracellular signaling molecules	Cdc42, Rac1, RhoE, IRS-2	[52-54]
Transporters	glut-1, MDR1, VDAC1	[55-57]
Membrane proteins	ANGPTL4, L1CAM, α5 integrin, CD151, CD24, CD147, Galectin-1, MUC1, Semaphorin 4D, Caveolin-1	[58-66]
Scaffold protein, cytoplasmic protein	HEF1, Liprin-α4	[67,68]
Matricellular proteins	CYR61, NOV	[69]
microRNAs	miR-210, miR-15b/16, miR-21, miR-372/373, miR-34a, miR-17/20a, miR-103/107, miR-126	[73-80]

From this review, it appears that different categories of targets can contribute to the metastatic process. Judging from the various steps involved in tumor metastasis, it is not surprising. However, it will need further investigation to figure out the exact mechanisms of how these targets contribute to metastasis. In addition, the interacting roles played by these targets to mediate metastasis will also require an integrated approach to combine molecular biology and systems biology in order to put all these players into a more comprehensive picture. Furthermore, novel hypoxia/HIF-1α-regulated targets that can mediate metastasis will need to be identified in order to complete the full picture of tumor metastasis induced by hypoxia/HIF-1α. Finally, elucidation of the functions of these targets mediating metastasis will provide the possibility of using these targets as therapeutic venues to improve the treatment of hypoxia-induced tumors.

## Abbreviations

HIF-1: Hypoxia-inducible factor-1; EMT: Epithelial-mesenchymal transition; ARNT: Aryl hydrocarbon receptor nuclear translocator; EWS-FLI1: Ewing Sarcoma-Friend leukemia virus integration 1; CDX2: Caudal type homeobox 2; SMAD7: SMAD family member 7; JMJD2B (KDM4B): Lysine-specific demethylase 4B; ACE: Angiotensin converting enzyme; CX3CR1: Chemokine (C-X3-C motif) receptor 1; IRS-2: Insulin receptor substrate 2; glut-1: Glucose transporter type 1; MDR1: Multidrug resistance protein 1; ID2: Inhibitor of differentiation/DNA binding 2; MMP: Matrix metalloprotease; LOX: Lysyl oxidase; ADAMTS1: A disintegrin and metalloproteinase with thrombospondin motifs 1; pdcd4: Programmed cell death 4; ANGPTL4: Angiopoietin-like 4; L1CAM: L1 cell adhesion molecule; RECK: Reversion-inducing-cysteine-rich protein with kazal motifs; STAT3: Signal transducer and activator of transcription 3; DAPK: Death-associated protein kinase; KLF4: Kruppel-like factor 4; Hsulf-1: sulfatase 1; XPA: Xeroderma pigmentosum, complementation group A; SF/HGF: Scatter factor/hepatocyte growth factor.

## Competing interests

The authors declare that they have no competing interests.

## Authors’ contributions

KJW completed the final draft and revision of the manuscript. Both authors read and approved the final manuscript.
